# Measuring Responsibility and Cooperation in Learning Teams in the University Setting: Validation of a Questionnaire

**DOI:** 10.3389/fpsyg.2018.00326

**Published:** 2018-03-13

**Authors:** Benito León-del-Barco, Santiago Mendo-Lázaro, Elena Felipe-Castaño, Fernando Fajardo-Bullón, Damián Iglesias-Gallego

**Affiliations:** ^1^Department of Psychology and Anthropology, University of Extremadura, Cáceres, Spain; ^2^Department of Didactics of Music, Plastic and Body Expression, University of Extremadura, Cáceres, Spain

**Keywords:** responsibility, cooperation, team efficacy, cooperative learning, university, students

## Abstract

Cooperative learning are being used increasingly in the university classroom, in order to promote teamwork among students, improve performance and develop interpersonal competences. Responsibility and cooperation are two fundamental pillars of cooperative learning. Team members’ responsibility is a necessary condition for the team’s success in the assigned tasks. Students must be aware that they depend on each other and should make their maximum effort. On the other hand, in efficient groups, the members cooperate and pool their efforts to achieve the proposed goals. In this research, we propose to create a *Questionnaire of Group Responsibility and Cooperation in Learning Teams (CRCG)*. Participants in this work were 375 students from the Faculty of Teacher Training of the University of Extremadura (Spain). The CRCG has very acceptable psychometric characteristics, good internal consistency, and temporal reliability. Moreover, structural equation analysis allowed us to verify that the latent variables in the two factors found are well defined and, therefore, their assessment is adequate. Besides, we found high significant correlations between the Learning Team Potency Questionnaire (CPEA) and the total score and the factors of the CRCG. This tool will evaluate cooperative skills and offer faculty information in order to prepare students for teamwork and conflict resolution.

## Introduction

### A Brief Description of Cooperative Learning Research

Most research on cooperative learning focused on the analysis of the consequences and outcomes of its application on academic, social, and affective variables (Johnson et al., 1981, 1983; Kromrey and Purdom, 1995; Kennett et al., 1996). With regard to the academic variables, cooperative learning increases the performance and productivity of participants (Johnson et al., 1981). Students improve the quality of their learning strategies and develop information-processing strategies, favoring critical and constructive thinking while promoting their capacity of communication and expression (Solsona, 1999).

Cooperative learning produces very interesting pedagogical results: intrinsic motivation, positive attitudes toward the subject, self-esteem, social support, group cohesion, participation, etc. (Cava, 1998; Del Caño and Mazaira, 2002). In general, situations of cooperative learning are more dynamic, attractive, and fun, they grant more responsibility and power to the students concerning their learning, while increasing their perceptions of autonomy and competence. At the affective and social levels, cooperative learning techniques influence motivation and contribute powerfully to the development and improvement of cooperative skills (Ovejero, 1993). Cooperative learning creates more interpersonal attraction among the students, leading to more positive attitudes toward classmates who are different (Johnson and Johnson, 1990). Some studies have analyzed the effect of cooperative learning on the attitudes of autochthonous students toward immigrants (León et al., 2009), and school bullying (León et al., 2012).

A few investigations have focused mainly on issues related to the efficacy of cooperative learning and the mediating mechanisms involved. The goal of these investigations revolves around two axes. The first one is the nature and quality of the interactive process (Johnson et al., 1990; Bennet and Dunne, 1991). The second axis refers to prior factors that condition the efficacy of cooperative learning (O’Donnel et al., 1990; Rewey et al., 1992; León et al., 2004; León, 2006). A series of studies shows that certain individual features may influence the positive results of cooperative learning. Among others, individual differences in verbal skill and cognitive style have been studied (Rewey et al., 1992). It has also been confirmed that cooperative learning may be more effective in people with high cognitive induction skills, extroversion (Hall et al., 1988), and social orientation.

In the university setting, we note the work of León et al. (2004), which analyzes how participants’ characters—introvert, extrovert, independent, gregarious, shy—affects the success and failure of cooperative learning. León (2006) corroborates the influence of social skills and group dynamics training on achievement and the interactive processes in cooperative learning situations. The more the resources of social interaction are consolidated in the group, the higher the achievement and efficacy of cooperative systems. Other researchers, too emphasize the importance of preparing students to make the most of cooperative learning situations (Johnson and Johnson, 1982; Webb, 2009; Buchs et al., 2016).

### Cooperative Learning in the University

The adaptation of the European Higher Education Area (EHEA), which started in 1999, involves important methodological changes, a new allocation of meanings to the teaching and learning tasks (González and Raposo, 2008). For Palacios (2004), the teaching processes and the teacher’s work in presential teaching are no longer as interesting as the learning processes by which the student attains the proposed goals in each subject. According to this new learning-focused approach, new methodologies like cooperative learning, which facilitate and reinforce students’ autonomous learning, are needed.

Methods of collaborative and cooperative learning or other forms of group learning are being used increasingly in the classroom in order to promote teamwork among students, improve performance and learning or develop interpersonal competences (Gaudet et al., 2010; Kirschner et al., 2011; Mendo et al., 2016; León et al., 2017).

Cooperative learning at university is an efficacious methodology to develop transversal competences, such as a critical sense and tolerance, transcending the strictly academic aspect and facilitating the practice of habits of cooperation, solidarity, and teamwork. The latter are key aspects in most business organizational schemes. According to Colás (1993), between 70 and 80% of jobs require a complex coordination of ideas and efforts, a capacity that can only be experienced and learnt through situations of cooperative learning.

However, despite its advantages, the application of cooperative learning in the university classroom is not without problems. The organizational structure, the competitive climate, the few social objectives related to higher education courses, and the emphasis on theoretical concepts for achieving academic success, do not favor its application (Darnon et al., 2009; Buchs et al., 2016). The development of social competences has traditionally been perceived as secondary and not particularly relevant in higher education (Gismero, 2000; Gillies, 2008).

Teamwork is not always received positively by students (Burdett, 2006; Payne and Monk-Turner, 2006; Hammar Chiriac, 2014). It is not enough to assign a task to students and tell them to work together. In order to introduce teamwork in the classroom, the following aspects are essential: the teacher’s preparation in the use of methodologies favoring peer cooperation, students’ training in teamwork (Beigi and Shirmohammadi, 2012), the team’s social skills (Rodríguez and Ridao, 2014; León et al., 2015), task design (Hijzen et al., 2007), and the team’s beliefs about its efficacy and performance, interdependence, conflicts, etc. (León et al., 2017). When these elements are not taken into account, unsatisfactory work experiences may discourage people from teamwork.

Team members’ responsibility is a necessary condition for the team’s success in the assigned tasks. Students must become aware that they depend on each other and should put out maximum effort. According to Johnson and Johnson (1987b), cooperative learning groups are based on positive interdependence among the group components. The goals are structured so that the students are not only interested in their own effort and performance but also in others’ performance. There is clear individual responsibility; all the team members share the responsibility for learning. Smith (1996) underlines positive interdependence and responsibility as essential elements for the success of a cooperative learning group. Students are successful if the team is successful. Each team member commits to carrying out his or her part of the work and the team is considered responsible for achieving the goals.

Many authors underline the importance of group members’ cooperative skills and the group’s maturity (Johnson et al., 1990). In fact, according to Pallarés (1993), productivity and group learning depend on the maturity attained when growing. According to this author, in efficient groups, the members cooperate, joining forces to achieve the proposed goals. The idea is to collaborate, not compete; along these same lines, Bruffee (1995) notes that cooperation implies working together and providing mutual support to reach goals. According to Ovejero (1990), it is necessary to take into account a series of aspects to perform a task that is truly cooperative: individual responsibility and cooperative skills.

### The Present Research

Diverse investigations have focused on the assessment of some of the variables related to cooperation in learning teams teamwork, such as the team’s social skills (León et al., 2015), the team’s potency (León et al., 2017), the preference or appraisal of the teamwork experience (Gottschall and García-Bayonas, 2008; Alford et al., 2014; Mendo et al., 2017), assessment and work environment (Beigi and Shirmohammadi, 2012), motivation (Ibarra and Rodríguez, 2007; Järvelä et al., 2010), the quality of the product and process, the classmates’ support, interdependence, and frustration (Nausheen et al., 2013).

Nevertheless, we believe that the organization, coordination, cohesion, solidarity, commitment, conflicts or the capacity to take criticism on board are some of the most relevant variables that determine whether work teams have been responsible and cooperative. Responsibility and cooperation are two fundamental pillars of cooperative learning. Individual learning as the result of teamwork will be better if there has been cooperation and responsibility (Johnson and Johnson, 1987a; Johnson et al., 1998).

It is important to design instruments to assess and delimit these variables in the university setting in situations of cooperative learning. Del Canto et al. (2009) point out as a source of team conflict the lack of responsibility, which translates into ‘*Free Rider’* attitudes, that is, students who do not do their share of the work and always have the perfect excuse, negatively influencing the team’s motivation, productivity, and efficiency (Kerr and Bruun, 1983).

Social loafing is a phenomenon that occurs in groups and consists of a reduction in the effort and motivation of the participants when they are collectively responsible for carrying out a task (Hardy and Latané, 1988). This phenomenon most usually occurs when one or more of the participants believes that the other members of the group will do the work or when they believe it is difficult to evaluate their contribution (Karau and Williams, 1993). In the context of teamwork, evaluating the responsibility and cooperation will provide the teacher with information with which to resolve the conflicts arising from this *Free Rider behavior*.

Furthermore, when hiring, companies are demanding professionals who, in addition to a technological profile or a specialized training and professional experience, have basic and cooperative skills to be able to work in multidisciplinary teams within different environments. Having an instrument that evaluates such cooperative skills will provide faculty with information to train his students in teamwork abilities and conflict resolution.

In this research, our aim is to build an instrument to evaluate the responsibility and cooperation, determine its structure and analyze its psychometric characteristics. The questionnaire will provide the teachers and researchers with diagnostic information concerning the cooperative, or collaborative, learning teams, or other forms of group learning in the university, information which will transcend the evaluation of the product and permit an analysis of the process and the working of the team.

## Materials and Methods

### Participants

In this work, the participants were 375 students (66% females and 44% males), between 18 and 44 years of age. The mean age was 21.3 years (*SD* = 4.6). The participants were selected randomly using a cluster sampling, where 6 classes were randomly selected from a total of 16 (1680 licentiate students studying Primary and Social Education) of the Faculty of Teacher Training of the University of Extremadura (Spain).

It is important to underline why we selected students from Primary Education and Social Education for our study. The academic guidelines of these degrees present a large quantity of contents and activities related to teamwork as well as the competences related to such contents and activities, which the students should carry out throughout their training process. A high percentage is also assigned to their assessment, so we can get an idea of the importance of teamwork for the participants in the study.

### Instruments

The *“Cuestionario de Responsabilidad y Cooperación Grupal en Equipos de Aprendizaje”* (CRCG [Questionnaire of Group Responsibility and Cooperation in Learning Teams]).

This questionnaire was designed taking as the starting point some of the most common behavior patterns that the classic research has identified as determinant in the question of teamwork cooperation (Johnson and Johnson, 1987a; Smith, 1996; Johnson et al., 1998).

The CRCG is made up of 14 items on a Likert type scale of 5 points, from 1 (never) to 5 (always) grouped in two dimensions: That of Responsibility (8 items) evaluates to what extent a team is capable of fulfilling the team’s aims and obligations effectively. The dimension of Cooperation (6 items) is related to the evaluation of certain factors which allow the effort to be pooled in order to achieve a particular result from the interaction with other people.

The questionnaire’s unit of study is the participation of the team as a whole and this allows intra-group information to be obtained from the opinion that each participant has concerning the rest of the team as regards the frequency with which the team mates generally organize, coordinate, give input, fulfill their part of the work, positively resolve conflicts, are cohesive, supportive, accept criticism, or make an effort when carrying out group activities in the different subjects.

In a context where the responsibility and cooperation of each team member influences the team’s effectiveness, as well as the group’s qualification, the CRCG allows a collective average score to be obtained for the different members of the group, avoiding such biases in the evaluation as falsification and deception in the self reporting (Dunning et al., 2004) or the evaluation of team mates based on affective-emotional relationships (Martín, 2010).

The *Cuestionario de Potencia de Equipos de Aprendizaje (CPEA* [Learning Team Potency Questionnaire]; León et al., 2017). The CPEA assesses students’ perception of their work team’s capacity to successfully perform the activities in the different subjects. It is made up of 8 Likert-type items with 10 response options ranging from 1 (*completely disagree*) to 10 (*completely agree*). The CPEA has two factors: the first factor, *Confidence* (4 items), assesses students’ expectations about their own team’s efficacy. The second factor, *Performance* (4 items), assesses students’ perception of their team’s capacity to successfully perform a series of academic tasks. Example items are: F1: “It is easy for my team to carry out any activity proposed in the different subjects”; F2: “The teamwork carried out by my team is very high quality.” The alpha indexes (α = 0.90) and composite reliability (CR = 0.95) show that the CPEA presents good global reliability and average variance extracted (AVE = 0.70). The two factors of the questionnaire present adequate reliability and an AVE > 0.70 in both factors [F1 (α = 0.85, CR = 0.91, AVE = 0.71); F2 (α = 0.81, CR = 0.92, AVE = 0.73)].

### Procedure

This research is included in a larger Project called “Skills Development Working in Cooperative Teams in Educational Settings,” it was approved by the Bioethical Committee of the University of Extremadura.

Firstly, the coordinator previously explained the study and asked the participants if they had any questions. All students gave written informed consent to take part in the investigation. In order to ensure the anonymity of the responses, a code number was assigned to each participant, thus guaranteeing the confidentiality of the data and their exclusive use for research purposes. We followed the ethical guidelines of the American Psychological Association (2009). Subsequently, in order to establish temporal reliability, following the same procedure 20 days later, 125 of the participants again completed the CRCG.

### Data Analysis

Initially, for the development and analysis of the psychometric characteristics of the CRCG, a principal components exploratory factor analysis (EFA) was carried out, after which we confirmed the factor structure found with a confirmatory factor analysis (CFA). To determine the invariance by gender of the obtained model, we performed a multi-group analysis. The stability and factor loadings of the model were established with the bootstrap method. Subsequently, we calculated correlations and comparisons of means to establish convergent and nomological validity. The reliability of the CRCG was calculated using Cronbach’s alpha, the composite reliability coefficients and the AVE. The EFA and correlations were performed with the SPSS-21 program, while for the CFA, the AMOS-21 program was used.

## Results

The original sample (*n* = 375) was divided into two randomly extracted subsamples (*n*_1_ = 175 and *n*_2_ = 200). The first one (*n*_1_) was used to carry out the EFA and the second (*n*_2_) as a validation sample for the CFA. Both subsamples are equivalent as regards age [*t*(373) = 0.439, *p* = 0.446] and gender [χ^2^(1) = 0.029, *p* = 0.864].

### Factor Analysis of the CRCG

After discarding those items that had corrected homogeneity indices lower than 0.40 (e.g., *My teammates treated each other with respect and were polite; My teammates took the division of responsibilities into account*), the Kaiser-Meyer-Olkin measure of sample adequacy yielded a value of 0.924. Bartlett’s sphericity test was significant (χ^2^ = 1834.98, *p* ≤ 0.001). Both the KMO and Bartlett values indicate that the data are adequate and can yield interesting conclusions and, according to the eigenvalue > 1 criterion, reveal the existence of two factors that jointly explain 62% of the total variance.

We used principal components with oblimin rotation to extract the factors. The goal is to find a set of components that explain the greatest possible amount of the total variance of the original variables (**Table 1**). The first factor, which we call *“Responsibility,”* explains 53% of the variance and refers to each team member’s perception of the remaining teammates with regard to their responsibility in the cooperative activities. The second factor, *“Cooperation,”* explains 9% of the variance and assesses the degree of cooperation in the team tasks. The internal consistency of the questionnaire, as measured by the Cronbach alpha index, was 0.931. This is very high and quite acceptable for the factors Responsibility (α = 0.912) and Cooperation (α = 0.847). With regard to the temporal reliability, the test–retest reliability coefficient (*r* = 0.870, *p* < 0.001) indicated a high stability of the scores. Regarding temporal reliability, the correlation between the scores was 0.810 (*p* < 0.001).

**Table 1:** Factor analysis of the Questionnaire of Group Responsibility and Cooperation in Learning Teams (CRCG) principal components with oblimin rotation.

Items of the instrument	*M*	SD	Commonalities	Factor 1	Factor 2
(1) My teammates have put out maximum effort	3.98	0.841	0.755	0.858	
(2) My teammates have worked hard on the team	3.96	0.925	0.696	0.814	
(3) My teammates have performed well as a work team	4.10	0.845	0.679	0.805	
(4) My teammates have behaved responsibly	4.03	0.776	0.659	0.804	
(5) My teammates have worked responsibly so the group will reach the goals and perform the tasks	4.09	0.818	0.702	0.803	
(6) My teammates have organized and coordinated themselves efficiently	4.11	0.815	0.673	0.777	
(7) My teammates have prepared their share of the work efficaciously	3.95	0.832	0.564	0.732	
(8) My teammates have contributed important information to the group	4.07	0.719	0.482	0.687	
(9) My teammates have encouraged the others	3.85	0.865	0.611		0.774
(10) My teammates have positively solved the conflicts and problems in the group	4.17	0.769	0.593		0.767
(11) My teammates have accepted criticism and suggestions positively	3.85	0.810	0.560		0.746
(12) My teammates have acted with solidarity and a high degree of cohesion.	4.11	0.754	0.600		0.711
(13) My teammates have collaborated simultaneously in the performance of the tasks	4.12	0.804	0.558		0.618
(14) My teammates have cooperated with each other	4.30	0.783	0.604		0.610
Percentage of explained variance (Total 62%) Alpha (Total 0.931)				53%	9%
				0.912	0.847

### Confirmatory Analysis of the CRCG

The CFA was performed with the second subsample (*n*_2_ = 200). An ideal sample for confirmatory analysis should include between 150 and 200 subjects (Kline, 2005). According to Henson and Roberts (2006), in the psychometric study of a questionnaire, it is a good practice to confirm the factor structure found in EFA with CFA. With the EFA, it is possible to determine the number of factors, but not which items are related to each factor or the relationships between the factors. The CFA is used to demonstrate the validity of the factorial structure previously obtained with the EFA and, therefore, the validity of the inferred theoretical deductions (Arruda et al., 1996). Given that the Exploratory Factorial Analysis (AFE) was not devised for proving hypotheses or theories, the data are subjected to a Confirmatory Factorial Analysis (AFC) to check the following prior hypotheses: (a) the number of factors, (b) which factors are related or are independent, and (c) with which factor or factors are each of the variables related.

In order to check these hypotheses, three models are tested. The first one to be examined is that in which the existence of a single factor of the first order is postulated, in which case, all the items are related with this factor. Then, to check the hypothesis that allows us to verify the relationships between the factors, the two independent factor model and the two related factor model are tested.

In order to use the maximum likelihood method (Jöreskog and Sörbom, 1996) to perform the estimations, the assumptions of linearity and normal distribution of all the variables of the model should be met. The residual dispersion graphics showed linearity among the estimated variables. To determine whether the sample meets the normality criterion, we examined atypical values by applying Mahalanobis’ distance, using the *Tests for normality and outliers* option of the AMOS program. After eliminating some atypical scores, the data of the sample met the normality criterion.

**Table 2** presents the goodness-of-fit indexes, considering three models: a one-factor model, a model with two independent factors, and a model with two correlated factors.

**Table 2:** Goodness-of-fit indexes of the proposed models.

Models	χ^2^	CMIN/*df*	CFI	TLI	RMSEA	SRMR
(1) Sole factor	*p* ≤ 0.001	2.465	0.935	0.924	0.086	0.048
(2) Independent factors	*p* ≤ 0.001	4.795	0.833	0.803	0.138	0.342
(3) related factors	*p* ≤ 0.001	1.976	0.958	0.949	0.060	0.041

All the models present a significant chi square value (*p* < 0.01) but as chi square tends to be statistically significant with large samples, from a practical perspective, it is more appropriate to take into account the magnitude of the value of chi square or of CMIN/*df* than the level of statistical significance: large values correspond to a poor fit and small values to a good fit.

**FIGURE 1:**
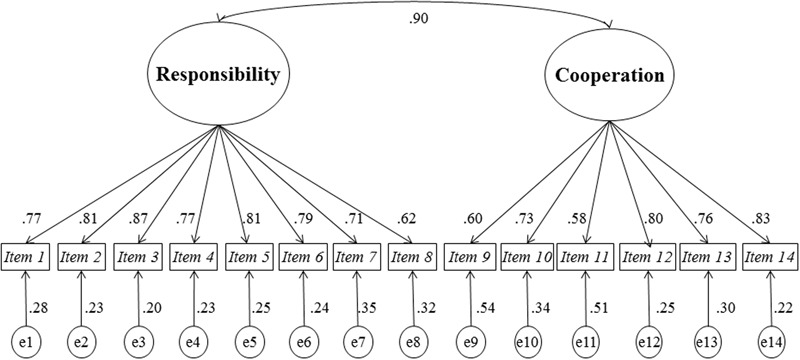
Model of the CRCG (*Questionnaire of Group Responsibility and Cooperation in Learning Teams*) with two related factors.

We discarded the 1-factor model and the model of 2 independent factors. The CFI and TLI fit indexes should be higher than or equal to 0.95, a value that these models did not reach. Nor were values of the RMSEA and SRMR indicators reached, which should be lower than 0.08 and 0.10, respectively. The model with two related factors presented the best fit-index values (**Figure 1**).

The results of the model indicated that factors “Responsibility” and “Cooperation” are correlated (Φ = 0.90). On the other hand, the indicators of the latent factors revealed factor loadings ranging between λ = 0.62 and λ = 0.87 for the factor “Responsibility” and between λ = 0.58 and λ = 0.83 for the factor “Cooperation.” This indicates that the factors are well defined and, therefore, their assessment was adequate.

The model with two correlated factors shows evidence of the reliability of the questionnaire, with values higher than 0.50 for AVE, and values of 0.85 for CR (Global reliability: AVE = 0.57, CR = 0.95; Factor “Responsibility”: AVE = 0.60, CR = 0.92; Factor “Cooperation”: AVE = 0.52, CR = 0.87).

To show that the values obtained in the factor loadings are not conditioned by a single sample and are significant, we calculated a 95% confidence interval (CI) for these values using the bootstrap method, considered the most general and classic resampling method (Efron, 1979; Efron and Tibshirani, 1993). This method allows creating a large number of samples with replacement with the same data and calculating for each sample the value of the statistic studied. As shown in **Table 3**, using a total of 1000 samples, we obtained means for the values of the factor loadings very close to the values found in the confirmatory analysis. However, the values of the factor loadings fall between the upper and lower limits of the 95% CI and, therefore, all of them are significant.

**Table 3:** Bootstrap method, 1000 samples with 95% CI.

Factors CRCG	Items	Factor loadings	Mean 1000 samples	Lower limit	Upper limit	*p*
Factor 1: Responsibility	CRCG1	0.769	0.771	0.713	0.818	0.015
	CRCG2	0.814	0.814	0.751	0.869	0.011
	CRCG3	0.866	0.865	0.823	0.904	0.009
	CRCG4	0.775	0.774	0.722	0.825	0.009
	CRCG5	0.812	0.812	0.748	0.862	0.013
	CRCG6	0.794	0.792	0.741	0.839	0.009
	CRCG7	0.710	0.710	0.628	0.776	0.013
	CRCG8	0.619	0.617	0.519	0.699	0.011
Factor 2: Cooperation	CRCG9	0.600	0.595	0.491	0.670	0.012
	CRCG10	0.726	0.730	0.626	0.794	0.021
	CRCG11	0.584	0.584	0.473	0.660	0.016
	CRCG12	0.801	0.799	0.735	0.851	0.010
	CRCG13	0.755	0.753	0.688	0.821	0.009
	CRCG14	0.830	0.826	0.764	0.876	0.007

### Analysis of Gender Invariance

We performed multi-group analysis to determine whether the model of two related factors was gender invariant, using a sample of 132 females and 68 males. **Table 4** presents the results obtained in the comparison of the different models. There were significant differences in the value of chi square between the unconstrained model and the model with measurement residuals (*p* ≤ 0.001). However, Cheung and Rensvold (2002) state that the difference in the ΔCFI values of the different nested models may be an indicator of factor structure invariance. When the difference of the CFI decreases by 0.01 or less, the unconstrained model is accepted, and the null hypothesis of invariance cannot be rejected. Therefore, the ΔCFI values found in the unconstrained model and the different models with invariance indicate that the factor loadings of questionnaire are equivalent for females and males.

**Table 4:** Multi-group analysis of gender invariance.

Models	χ^2^	*df*	χ^2^/*df*	Δχ^2^	Δ*df*	CFI	TLI	SRMR	RMSEA
Model 1	242.92	152	1.598	–	–	0.949	0.939	0.044	0.055
Model 2	276.16	164	1.684	33,236	12	0.937	0.930	0.051	0.059
Model 3	279.98	167	1.677	37,056	15	0.937	0.931	0.055	0.059
Model 4	299.74	181	1.656	56,821	29	0.933	0.933	0.061	0.058

*Model 1, unconstrained; Model 2, measurement weights. Model 3, structural covariances; Model 4, measurement residuals.*

### Nomological Validity

Nomological validity refers to the degree to which the relationships of a construct with other constructs that form part of or an entire theory or theories can be confirmed empirically; that is, whether the theoretical configuration of the data corresponds with the theoretical predictions of that configuration.

In this case, we related the CRCG scores with the factors of the *Learning Team Potency Questionnaire* (CPEA; León et al., 2017). We found high significant correlations between the CPEA (**Table 5**) and the total score and the factors of the CRCG. These high correlations indicate that when the students perceive responsibility and cooperation, their confidence in the group’s effectiveness increases.

**Table 5:** Pearson correlations between CPEA and CRCG factors.

CPEA team potency	CRCG		
	Total	F1 Responsibility	F2 Cooperation
Total	0.614^∗∗^	0.596^∗∗^	0.545^∗∗^
F1 Confidence	0.580^∗∗^	0.570^∗∗^	0.506^∗∗^
F2 Performance	0.577^∗∗^	0.553^∗∗^	0.522^∗∗^

*CPEA, Learning Team Potency Questionnaire; CRCG, Questionnaire of Group Responsibility and Cooperation in Learning Teams. ^∗∗^ The correlation is significant at the 0.01 level.*

## Discussion

This aim of this present study was to create and validate a questionnaire that would allow us to simply and quickly evaluate responsibility and cooperation in university learning groups.

Regarding our goal, we can state that the CRCG has very acceptable psychometric characteristics, good internal consistency and temporal reliability. The factor adequacy measures of Kaiser-Meyer-Olkin and Bartlett’s test confirm the suitability of factor analysis. The analysis carried out has shown the existence of the two solid and well defined factors on which we based the construction of the scales, which explain 62% of the total variance. The factor loadings of the items that define the two factors have values higher than 0.50. According to Costello and Osborne (2005), when a factor is defined by 4–5 items with loadings above 0.50, it is a solid factor with practical relevance.

Lastly, the questionnaire was submitted to CFA, testing the three different factor structures: the first one, made up of 14 items grouped into a sole factor, the second with two independent factors, and the third with two correlated factors. The values that presented the best fit were those of the model with two related factors. The correlations between the two factors are high and statistically significant (*p* < 0.01). As the perceived degree of responsibility increases in one factor, the degree of cooperation in the other factor also increases.

Structural equation analysis and the application of the bootstrap method allowed us to verify that the values of the factor loadings fell between the upper and lower limits of the 95% CI, and all of them were significant. Therefore, the latent variables in the two factors are well defined and adequately assessed, reaffirming the good psychometric characteristics of the scale.

Diverse investigations reveal that females usually score higher than males in social skills and cooperation (Bandeira et al., 2006). In general, women are more expressive than men and, in their investigations, authors like Lafferty (2004) and Tapia and Marsh (2006), among others, showed that women are more perceptive, display more empathy, and better recognize other people’s emotions. This would explain their more positive social interaction with others. To ensure that in future investigations of the CRCG, gender differences are due to real differences in the construct assessed and not to different psychometric responses to the items of the questionnaire (Cheung and Rensvold, 2002; Pedraza and Mungas, 2008), we conducted multi-group analysis to determine whether the model of two related factors was gender invariant. The results obtained confirm the equivalence of men and women in the perception of the assessed construct. The data support the equivalence of the factor structure of the CRCG as a function of gender.

In addition, the relations between the CRCG and the CPEA clearly indicate an association between group cooperative skills and the beliefs of the team members about the team’s capacity to be effective. The concept of team potency was originally defined by Guzzo et al. (1993) in reference to a group’s collective beliefs in its effectiveness, and it is an essential construct related to group motivation. Team potency is the most relevant variable to predict performance and group efficacy when compared with other variables such as group composition, interdependence, work design, and organizational setting (Campion et al., 1996).

In situations of cooperative learning, students become aware that depend on one another and they must make the maximum effort and cooperate. All the team members share the responsibility for learning. Each team member commits to carrying out his or her part of the work and the team is considered responsible for achieving the goals. When the students perceive their team’s responsibility and cooperation, they think that the team has worked more efficaciously and it stimulates cohesion and trust among the members. No doubt, these mechanisms of cooperation and responsibility will increase confidence in the efficacy of the team.

On another hand, although the CRCG presents sufficient evidence of validity and reliability, it is not exempt from limitations, such as the difficulty to generalize the results to other groups of university population, which compromises the external validity (population and ecological) of the questionnaire, or to establish greater evidence of convergent and discriminant validity. In addition, the design of the items for the CRCG does not allow the extraction of self-reports or individual evaluations of the behavior of the different group members. As commented above, although this does reduce some evaluation biases, it also impedes obtaining individual results from the different team members. It would, therefore, be interesting to validate different versions of the CRCG, as this would allow us to establish self-reporting or individual evaluations individuals so as to better understand how each member of the team functions specifically. As future lines of research, besides resolving these limitations, it would be of interest to validate the CRCG in a non-university population.

Lastly, based on the above, it can be concluded that the CRCG can help to understand the Responsibility and Cooperation in Learning Teams in the University Setting. Its application is simple and fast, and it can be useful as a diagnostic and/or predictive measure, allowing us to know the students’ attitudes toward teamwork in general or regarding a certain subject or material, and if necessary, to design actions to improve teacher practice in cooperative, collaborative learning, or other forms of group or team learning. We think that university professors should create the conditions to guarantee optimal responsibility and cooperation in cooperative learning teams. To achieve this outcome implies teachers’ effort and interest and accepting that their role not only determines good team functioning and goal achievement, but also the satisfaction of all the students who participate in the diverse teams. To achieve the many advantages of cooperative learning in the university classroom requires the teachers to carefully design a program and to perform interventions throughout the process to resolve conflicts, and subsequently to analyze the teamwork.

There are many evolutionary reasons for the members of an intelligent species to try to live in peace (Pinker, 2003). Many computer simulations and mathematical models have demonstrated that cooperation is profitable from an evolutionary viewpoint. Cooperation is a characteristic of the human being that differentiates us from other species. In fact, cooperation has allowed us to adapt constantly to new environmental situations, making human development possible.

## Author Contributions

BL-d-B and SM-L: analysis and interpretation of the data and also drafting the work. BL-d-B, SM-L, EF-C, FF-B, and DI-G: the conception and design of the work. All authors listed have made a substantial, direct and intellectual contribution to the work, and approved it for publication.

## Conflict of Interest Statement

The authors declare that the research was conducted in the absence of any commercial or financial relationships that could be construed as a potential conflict of interest.
